# Safety and Efficacy of Left Atrial Catheter Ablation in Patients with Left Atrial Appendage Occlusion Devices

**DOI:** 10.3390/jcm11113110

**Published:** 2022-05-31

**Authors:** Binhao Wang, Bin He, Guohua Fu, Mingjun Feng, Xianfeng Du, Jing Liu, Yibo Yu, Huimin Chu

**Affiliations:** Arrhythmia Center, Ningbo First Hospital, Ningbo 315000, China; wangbinhao0504@163.com (B.W.); socrates_he@126.com (B.H.); eagle1002@126.com (G.F.); fmj76@126.com (M.F.); drduxianfeng@126.com (X.D.); nblight6@126.com (J.L.); mubird@foxmail.com (Y.Y.)

**Keywords:** atrial fibrillation, catheter ablation, left atrial appendage occlusion

## Abstract

Background: Left atrial appendage occlusion (LAAO) is an alternative to oral anticoagulation for thromboembolic prevention in patients with atrial fibrillation (AF). Left atrial (LA) catheter ablation (CA) in patients with LAAO devices has not been well investigated. Here, we report on the safety and efficacy of LA CA in patients with nitinol cage or plug LAAO devices. Methods: A total of 18 patients (aged 67 ± 11 years; 14 males; 5 paroxysmal AF) with LAAO devices (nitinol cage, *n* = 10; nitinol plug, *n* = 8) and symptomatic LA tachyarrhythmias were included. Periprocedural and follow-up data were assessed. Results: A total of 20 LA CA procedures were performed at a median of 130 (63, 338) days after LAAO. The strategy of CA consisted of circumferential pulmonary vein isolation (*n* = 16), linear lesions (*n* = 14) and complex fractionated atrial electrogram ablation (*n* = 6). No major adverse events occurred periprocedurally. Repeated transesophageal echocardiography showed no device-related thrombus, newly developed peridevice leakage or device dislodgement. After a median follow-up period of 793 (376, 1090) days, four patients (22%) experienced LA tachyarrhythmias recurrence and two received redo LA CA. No patients suffered stroke or major bleeding events during follow-up. Conclusions: LA CA in patients with LAAO devices (either nitinol cages or nitinol plugs) seems to be safe and efficient in our single-center experience.

## 1. Introduction

Atrial fibrillation (AF) is the most common arrhythmia in the clinical setting and significantly increases the risk of stroke [1]. The majority of thrombi originate from the left atrial appendage (LAA) [2]. Oral anticoagulation (OAC) is recommended for nonvalvular AF patients with CHA_2_DS_2_-VASc scores ≥ 2 in males or ≥3 in females [1]. However, some patients have contraindications for long-term OAC (e.g., major bleeding events under OAC). Recently, left atrial appendage occlusion (LAAO) was proven to be noninferior to warfarin [3] and the nonvitamin antagonist oral anticoagulant (NOAC) [4] in stroke prevention. Left atrial (LA) catheter ablation (CA) is effective in maintaining sinus rhythm in patients with symptomatic AF [5]. However, the long-term influence of LA CA on stroke prevention remains unclear. Therefore, combining LA CA and LAAO in a single procedure has emerged as a successful strategy [6,7,8].

However, some patients may undergo LAAO first for stroke prevention. They may require LA CA for symptomatic LA tachyarrhythmias in the future. Data regarding this issue are limited. A small number of case reports [8,9,10] and case series [11,12,13,14,15] with small samples have been carried out to investigate the feasibility and efficacy of LA CA in patients with LAAO devices. However, most patients in prior studies were implanted with nitinol cage devices (e.g., Watchman). Investigations concerning LA CA in patients with nitinol plug devices (e.g., Amplatzer Cardiac Plug, ACP) are rare. We report on the safety and efficacy of LA CA for the treatment of LA tachyarrhythmias in patients with nitinol cage or plug LAAO devices.

## 2. Materials and Methods

### 2.1. Study Population

This was a retrospective, single-center study to assess the safety and efficacy of LA CA in 18 consecutive patients (aged 67 ± 11 years; 14 males; 5 exhibiting paroxysmal AF) with previously implanted LAAO devices (Watchman, *n* = 10; ACP, *n* = 4; LAmbre, *n* = 4) to treat symptomatic and drug-refractory LA tachyarrhythmias (AF, *n* = 15; atrial flutter (AFL), *n* = 1; atrial tachycardia (AT), *n* = 2) from March 2016 to October 2019 at Ningbo First Hospital. Patient characteristics were collected to calculate the individual CHA_2_DS_2_-VASc score [16] and HAS-BLED score [17]. Transesophageal echocardiography (TEE) was performed before LA CA to exclude LA thrombi and evaluate the LAAO devices for device-related thrombus (DRT) and peridevice leakage (PDL). Transthoracic echocardiography was also conducted to measure the LA diameter and left ventricular ejection fraction (LVEF). This study was approved by the Ethics Committee of Ningbo First Hospital and complies with the Declaration of Helsinki. Informed consent was obtained from all study participants.

### 2.2. LA CA Procedure

Antiarrhythmic drugs were stopped five half-lives before the procedure. Warfarin with a therapeutic international normalized ratio was continued uninterrupted, while NOACs were ceased 12~24 h preprocedurally. Patients were placed under deep sedation for LA CA. A decapolar diagnostic catheter was positioned in the coronary sinus through left femoral venous access. Double transseptal accesses were obtained for the placement of two sheaths via right femoral venous access. Intravenous heparin was administered prior to the first transseptal puncture with a target activated clotting time ≥ 350 s [1]. A circular mapping catheter and an irrigated tip ablation catheter were utilized for mapping and LA CA. Three-dimensional reconstruction of the LA and pulmonary veins (PV) was performed from the CT scan using electroanatomic mapping systems (CARTO, Biosense Webster, Diamond Bar, CA, USA; or Ensite NavX Verismo software, St. Jude Medical, St. Paul, MN, USA). A maximum temperature cutoff of 43 °C and maximum power cutoff of 35 W were chosen, with a catheter infusion rate of 17–25 mL/min. Circumferential pulmonary vein isolation (CPVI) was performed in patients with AF. Additional linear lesions and/or complex fractionated atrial electrograms (CFAEs) were targeted if necessary. For macroreentrant LA tachyarrhythmias, ablation of linear lesions was performed. During all procedures within the LA, catheter impedance was closely monitored to avoid device-related complications. After the procedures, all patients underwent TEE to exclude pericardial effusion and evaluate the LAAO devices. Periprocedural adverse events included thromboembolic events (stroke, transient ischemic attack (TIA), or systemic embolism), pericardial effusion, bleeding events, interference with the device and device dislodgement. Major bleeding was defined according to the BARC (Bleeding Academic Research Consortium) criteria (type 3 or higher) [18].

### 2.3. Follow-Up

Antiarrhythmic drugs and OACs were recommended for 3 months after the procedure. OAC was then discontinued and displaced by a recommended postimplant regimen for LAAO devices [1]. TEE follow-up was arranged to determine the presence of device dislodgement, DRT or newly developed PDL. Clinical follow-up for LA tachyarrhythmias recurrence was performed at 3, 6 and 12 months using 24 h Holter monitoring. Arrhythmia recurrence was defined as documented LA tachyarrhythmias (AF, AFL, and AT) that lasted at least 30 s after a 3 month blanking period. Thromboembolic events (stroke, TIA, or systemic embolism) and bleeding events (major or minor) were also recorded during follow-up.

### 2.4. Statistical Analysis

The study patients were divided into two groups according to the type of LAAO devices: a nitinol cage group (*n* = 10) and a nitinol plug group (*n* = 8). The nitinol cage group included patients with previously implanted Watchman devices. The nitinol plug group consisted of subjects with ACP or LAmbre devices. Continuous variables were expressed as the median (interquartile range). Categorical variables were expressed as absolute numbers (percentages). Continuous variables were compared using the Mann–Whitney U test. Categorical variables were compared using the chi-square test or Fisher’s exact test where appropriate. Survivor functions were estimated using the Kaplan–Meier method to assess the cumulative event-free curves of LA tachyarrhythmias for each group and statistically evaluated using a log-rank test of trend. Statistical analyses were performed with SPSS 19.0 (IBM, Armonk, NY, USA), and a *p* value < 0.05 was considered statistically significant.

## 3. Results

### 3.1. Patient Characteristics

Patient characteristics are displayed in Table 1. The median CHA_2_DS_2_-VASc and HAS-BLED scores were 4.5 (3, 6) and 3 (2.5, 4), respectively. Five patients (28%) had prior LA CA. The numbers of patients with prior stroke/TIA and bleeding were 16 (89%) and 7 (39%), respectively. The characteristics were comparable between the nitinol cage group and the nitinol plug group.

### 3.2. Periprocedural Data

The periprocedural data are shown in Table 2. The median time from LAAO to CA was 130 (63, 338) days. Twenty procedures (index procedure, *n* = 18; redo procedure, *n* = 2) were performed. During the index procedure in patients with AF or AFL, CPVI was targeted in all patients (*n* = 16), followed by linear lesions (*n* = 10) and CFAE (*n* = 5). The patient with AFL was mitral isthmus-dependent and was terminated by linear ablation at the mitral isthmus. One patient’s case of AT was terminated by creating a linear lesion at the ridge between the left PV and LAA. Another patient with AT underwent prior ablation with CPVI, and all PVs were confirmed to be isolated. Two types of AT were detected and terminated by linear lesions (roof line plus anterior line). No LAA isolation was performed.

**Figure 1:**
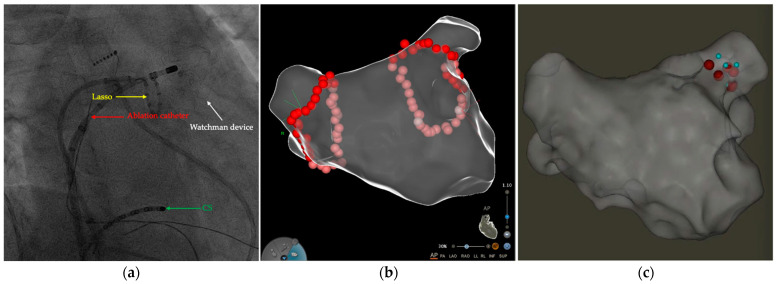

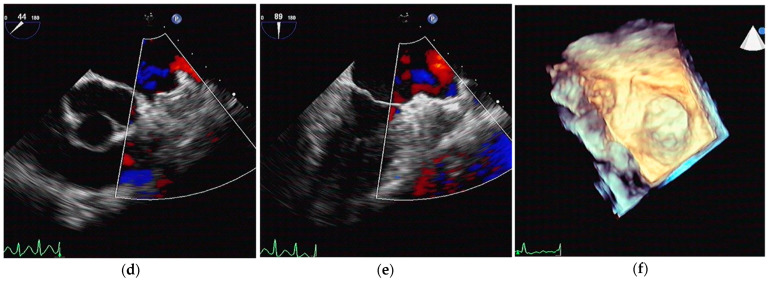
The LA CA procedure (index and redo) and TEE follow-up in a patient with an implanted LAAO device. (**a**) Fluoroscopic image showed the position of Watchman device (white arrow), coronary sinus catheter (green arrow), circular mapping catheter (yellow arrow) and ablation catheter (red arrow); (**b**) during the index procedure for paroxysmal AF, CPVI was successfully performed; (**c**) during the redo procedure for AFL, the ridge between the LAA and left superior PV were targeted (red dots) to restore sinus rhythm; (**d**–**f**) TEE follow-up after LA CA showed no device dislodgement, PDL, or DRT. AF—atrial fibrillation; AFL—atrial flutter; CA—catheter ablation; CPVI—circumferential pulmonary vein isolation; DRT—device-related thrombus; LA—left atrium; LAAO—left atrial appendage occlusion; PDL—peridevice leakage; PV—pulmonary vein.

Two patients received redo ablation. One had AF recurrence 346 days after the index procedure. All PVs were still isolated, and linear lesions (roof line, posterior line, and superior vena cava line) and CFAE ablation were targeted to restore sinus rhythm. Another patient showed AFL recurrence 378 days after the index procedure. Activation mapping revealed localized reentry at the ridge between the LAA and left superior PV, and ablation at this region terminated the AFL (Figure 1).

The procedural time, ablation time, X-ray exposure time and X-ray exposure dose were similar between the two groups. At the end of all procedures, all PVs were successfully isolated, and bidirectional block was achieved at all lines that were applied. No thromboembolic events, pericardial effusion, major bleeding events, interference with the device and device dislodgement occurred periprocedurally. Two patients experienced minor bleeding events at the puncture site (one in the nitinol cage group and one in the nitinol plug group).

### 3.3. Follow-Up Results

Follow-up results are shown in Table 3. OAC therapy was prescribed for all patients (two with warfarin, eight with dabigatran and eight with rivaroxaban) for 3 months. After 3 months, five patients switched to dual antiplatelet therapy (100 mg/d aspirin plus 75 mg/d clopidogrel) until 6 months post-LAAO, followed by single antiplatelet therapy (100 mg/d aspirin or 75 mg/d clopidogrel) indefinitely. The remaining 13 patients beyond 6 months after the LAAO procedure changed to single antiplatelet therapy. Repeated TEE was performed in 14 patients (78%). No DRT, newly developed PDL or device dislodgement was documented.

**Figure 2:**
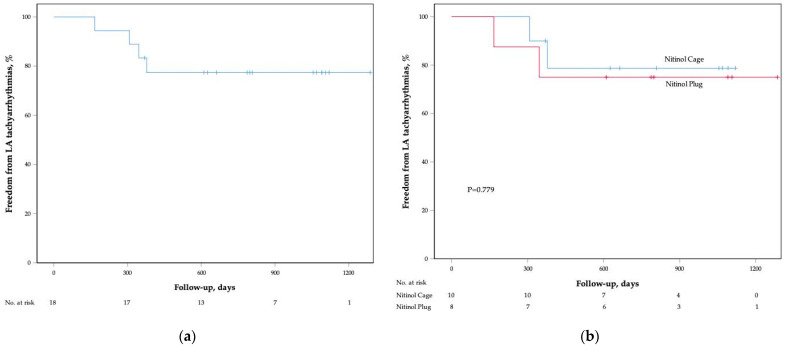
Kaplan–Meier cumulative event-free curves of LA tachyarrhythmias. (**a**) Total study population; (**b**) nitinol cage vs. nitinol plug. LA—left atrium.

After a median follow-up period of 793 (376, 1090) days, four patients (22%) experienced LA tachyarrhythmias recurrence (Figure 2a). The recurrence rates between the two groups showed no statistical significance (nitinol cage group, one AF and one AFL; nitinol plug group, two AF; Figure 2b). In addition, no patient suffered thromboembolic or major bleeding events. Only one patient in the nitinol cage group experienced gingival bleeding.

## 4. Discussion

We present a single-center study on the safety and efficacy of LA CA in patients with LAAO devices. To the best of our knowledge, the present investigation is the first case series report including patients with nitinol plug devices to date. Our main finding is that LA CA in patients with either nitinol cage or nitinol plug devices seems to be safe and efficient.

An animal study showed that endothelial cells covered the endocardial surface with smooth muscle cells within 45 days of Watchman device implantation [19]. In another canine study, there was complete coverage of the ACP atrial surface by stable mature neointima in the device and within the surface neointima within 90 days of implantation [20]. No occurrence of late Watchman device embolization has been reported in previous investigations thus far [12]. In addition, late device embolization of ACP devices was also rare, with only a few case reports [21,22]. The minimum timeframe from Watchman device implantation to LA CA ranged from 41 days to 190 days [11,12,13]. In the present study, the minimum time-points of LA CA following LAAO device implantation were 47 days and 63 days in the nitinol cage group and nitinol plug group, respectively. It remained unclear how soon after LAAO device implantation LA CA could be considered according to the limited data.

Impedance-measurement errors may occur during LA CA. A case reported indicated that delivery of radiofrequency energy near the ACP device resulted in automatic generator shut-off with impedance-measurement errors [8]. However, impedance-measurement errors did not occur in previous investigations or in our study. One patient with a Watchman device had thrombus formation at the site of the device after LA CA [11]. In a multicenter AF registry, a higher proportion (10%) of severe PDLs after LAA isolation in patients with Watchman devices was indicated [14]. However, there were no newly developed PDLs in most patients without LAA isolation. Cryoballoon ablation may be an optional choice to avoid the contact with the LAAO device. Huang et al. [15] performed PVI in patients with Watchman device implantation by cryoballoon ablation. The study showed that cryoballoon ablation was feasible and safe in patients with preexisting LAAO devices. The above findings may have resulted from the damage of endothelial tissue covering the endocardial surface of the LAAO devices caused by the application of LA CA. No LAA isolation was targeted in our study, and no new PDL or DRT was detected after LA CA. Therefore, several points must be addressed: (1) TEE and/or cardiac CT should be arranged before the LA CA procedure to evaluate the anatomic characteristics of the LAAO device and left PV. (2) During procedure, satisfied LA anatomic mapping should be performed to guide ablation. The ablation points at the ridge between LAA and left PV should be chosen nearer to the left PV side to avoid the contact with LAAO device. Additionally, close monitoring of catheter impedance should be performed to avoid device-related complications. (3) OAC may be necessary after the LA CA procedure due to the potential damage of endothelial tissue on the LAAO devices. Most patients in the prior investigations and in our study received OAC therapy for at least 3 months. (4) Follow-up TEE should be performed before discontinuation of OACs to assess the presence of DRT and PDL.

CPVI is the cornerstone for the treatment of AF [23]. However, additional linear lesions and CFAE ablation are often required for persistent AF [24]. In our study, CPVI plus additional ablation was successfully performed in all patients. One patient with AT for index CA and one with AFL for redo CA (both had a Watchman device) had their arrhythmias successfully terminated by targeting the ridge between the LAA and left PV. The LAA has been recognized as a potential arrhythmogenic source in AF [25]. The BELIEF trial demonstrated that LAA isolation improved long-term freedom from atrial arrhythmias without increasing complications in patients with longstanding persistent AF [26]. In the study by Turagam et al., 28% (17/60) of the study population had focal triggered activity from the LAA in patients with Watchman device implantation, and LAA isolation was achieved in 58% (10/17) [14]. LAA isolation is a challenging procedure in patients with LAAO devices. Theoretically, it should be more difficult in patients with nitinol plug devices covering the ostium of the LAA. The nitinol plug devices contain a proximal disc that extends outside the ostium of the LAA toward the LA ridge. Because of the overlap of this disc and the LA ridge, successful LAA isolation in these patients may be technically more difficult than for patients implanted with the nitinol cage devices. In addition, LAA isolation may result in newly developed PDLs, as mentioned above. Therefore, whether LAA isolation is necessary and feasible in patients with LAAO devices still needs further discussion.

No major periprocedural adverse events or thromboembolic or major bleeding events during follow-up occurred in our study. The complications in other studies were rare and mainly occurred at the site of puncture (e.g., hematoma and arteriovenous fistula) [12,13,14]. Additionally, none of the patients suffered major bleeding events or stroke during follow-up [12,13,14]. The rate of patients free from LA tachyarrhythmias during follow-up ranged from 42% to 83% in prior studies [12,13,14,15]. In the present research, 78% remained in sinus rhythm after a median follow-up period of 793 days. The difference in the recurrence rate in different studies may be attributed to the difference in patient characteristics and the limited number of patients. Therefore, LA CA for the treatment of LA tachyarrhythmias in patients with LAAO devices is likely to be safe and efficient. However, the safety and efficacy should be further assessed in the future in large study populations.

There are several limitations in our study. First, the study sample was relatively small. However, this is the first investigation including a case series with previously implanted nitinol plug devices. Second, all the procedures were performed using radiofrequency energy. The experience from the present investigation cannot be extended to other ablation energies (e.g., cryoablation and pulsed field ablation). Third, four patients refused to finish TEE follow-up after LA CA. Therefore, the presence or absence of DRT and newly developed PDL was unknown in those patients.

## 5. Conclusions

LA CA in patients with prior implanted LAAO devices (either nitinol cages or nitinol plugs) seems to be safe and efficient in our single-center experience. Further multicenter investigations with a large study population are needed to prove this finding in the future.

## Figures and Tables

**Table 1:** Baseline characteristics.

Variable	Total	Nitinol Cage	Nitinol Plug	*p* Value
*n*	18	10	8	-
Age, years	70 (60, 74)	64 (57, 78)	71 (68, 74)	0.450
Male, *n* (%)	14 (78)	9 (90)	5 (63)	0.275
Type of arrhythmias, *n* (%)				
Paroxysmal AF	5 (28)	3 (30)	2 (25)	1.000
Persistent AF	10 (56)	6 (60)	4 (50)	1.000
AFL	1 (6)	0	1 (13)	0.444
AT	2 (11)	1 (10)	1 (13)	1.000
CHA_2_DS_2_-VASc score, points	4.5 (3, 6)	4 (3, 6)	4.5 (4, 6)	0.752
HAS-BLED score, points	3 (2.5, 4)	3 (2, 4)	3 (3, 5)	0.483
Prior stroke/TIA, *n* (%)	16 (89)	10 (100)	6 (75)	0.183
Prior bleeding, *n* (%)	7 (39)	5 (50)	2 (25)	0.367
Prior LA CA, *n* (%)	5 (28)	3 (30)	2 (25)	1.000
LA diameter, mm	42 (39, 45)	41 (36, 47)	43 (40, 45)	0.532
LVEF, %	64 (63, 67)	65 (63, 68)	64 (63, 67)	0.788

AF—atrial fibrillation; AFL—atrial flutter; AT—atrial tachycardia; CA—catheter ablation; LA—left atrium; LVEF—left ventricular ejection fraction; TIA—transient ischemic attack.

**Table 2:** Periprocedural data.

Variable	Total	Nitinol Cage	Nitinol Plug	*p* Value
No. of LA CA	20	11	9	-
Strategy of CA, *n* (%)				
CPVI	16 (80)	9 (82)	7 (78)	1.000
Linear lesions	14 (70)	6 (55)	8 (89)	0.157
CFAE	6 (30)	3 (27)	3 (33)	1.000
Procedural time, min	119 (89, 130)	112 (99, 126)	122 (79, 131)	0.894
Ablation time, min	34 (23, 46)	31 (20, 39)	40 (25, 52)	0.328
X-ray exposure time, min	5.0 (4.0, 6.1)	5.0 (3.8, 6.1)	4.9 (4.1, 6.0)	1.000
X-ray exposure dose, mGy	25 (20, 36)	23 (19, 40)	28 (24, 34)	0.534
Complications, *n* (%)				
Stroke/TIA	0	0	0	1.000
Systemic embolism	0	0	0	1.000
Pericardial effusion	0	0	0	1.000
Major bleeding events	0	0	0	1.000
Minor bleeding events	2 (10)	1 (9)	1 (11)	1.000
Interference with device	0	0	0	1.000
Device dislodgement	0	0	0	1.000

CA—catheter ablation; CFAE—complex fractionated atrial electrogram; CPVI—circumferential pulmonary vein isolation; LA—left atrium; TIA—transient ischemic attack.

**Table 3:** Follow-up results.

Variable	Total	Nitinol Cage	Nitinol Plug	*p* Value
*n*	18	10	8	-
Recurrent LA tachyarrhythmias, *n* (%)	4 (22)	2 (20)	2 (25)	1.000
Redo ablation, *n* (%)	2 (11)	1 (10)	1 (13)	1.000
Stroke/TIA/systemic embolism, *n* (%)	0	0	0	1.000
Major bleeding events, *n* (%)	0	0	0	1.000
Minor bleeding events, *n* (%)	1 (6)	1 (10)	0	1.000
TEE follow-up, *n* (%)	14 (78)	8 (80)	6 (75)	1.000
DRT	0	0	0	1.000
Newly developed PDL	0	0	0	1.000
LAAO device dislodgement	0	0	0	1.000
OAC therapy, *n* (%)				
Warfarin	2 (11)	2 (20)	0	0.477
Dabigatran	8 (44)	4 (40)	4 (50)	1.000
Rivaroxaban	8 (44)	4 (40)	4 (50)	1.000

DRT—device-related thrombus; LA—left atrium; LAAO—left atrial appendage occlusion; OAC—oral anticoagulation; PDL—peridevice leakage; TEE—transesophageal echocardiography; TIA—transient ischemic attack.

## Data Availability

The data used to support the findings of this study are available from the corresponding author upon reasonable request.
